# Two cases of multiple ossifying fibromas in the jaws

**DOI:** 10.1186/1746-1596-9-75

**Published:** 2014-03-28

**Authors:** Ting-Ting Wang, Ran Zhang, Lin Wang, Yan Chen, Qing Dong, Tie-Jun Li

**Affiliations:** 1 Department of Oral Medicine, Hebei United University, School and Hospital of Stomatology, Tangshan, 82 South Construction Road, Hebei 063000, Lubei District, PR China; 2 Department of Oral Pathology, Peking University School and Hospital of Stomatology, 22 South Zhongguancun Avenue, Haidian District, Beijing 100081, PR China; 3 Central Laboratory, Peking University School and Hospital of Stomatology, Beijing, China; 4 National Engineering Laboratory for Digital and Material Technology of Stomatology, Beijing, China

**Keywords:** Multiple ossifying fibroma, HPT-JT, Fibrous dysplasia, *GNAS* gene, *HRPT2* gene, Osseous dysplasia

## Abstract

**Background:**

The clinicopathologic characteristics of multiple ossifying fibroma (OF) are unclear due to the condition’s rarity, making diagnosis challenging. Sporadic multiple OFs must be distinguished from hyperparathyroidism-jaw tumour syndrome (HPT-JT) related OF and other fibro-osseous lesions.

**Methods:**

Multiple OF cases were identified from ossifying fibroma cases. Clinical data including age, sex, anatomic site, radiographic features, clinical impression, treatment and available follow-up data as well as serum calcium, phosphorus, and parathyroid hormone (PTH) were recorded. *GNAS* and *HRPT2* genetic mutations were examined in the two present cases. Case reports of sporadic multiple ossifying fibroma and HPT-JT-related OF were also reviewed.

**Results:**

The two present cases were confirmed as sporadic multiple OF, with no genetic *GNAS* and *HRPT2* mutations found. The incidence of sporadic multiple ossifying fibroma was 2.0% (2/102). The total 18 sporadic multiform OF cases were characterized as followed: 13 (72.2%) female; 5 (27.8%) male; mean age 28.6 years; 2/16 (11.1%) cases only in the mandible; 4/18 (22.2%) cases only in the maxilla; and 12/18 (66.7%) cases in both the maxilla and mandible. Radiographically, the lesions were radiolucent in 5/18 (27.8%) cases and mixed density in 13/18 (72.2%) cases. Along with 24 cases of HPT-JT related OF were reviewed, sixteen (66.7%) patients were diagnosed with a single lesion, and 8 patients (33.3%) were diagnosed with multiple jaw lesions.

**Conclusions:**

Sporadic multiple OFs are very rare, but must be distinguished from HPT-JT related OF. We strongly recommend that patients diagnosed with multiple ossifying fibromas receive serum PTH testing and mutation screening of *HRPT2*.

**Virtual slides:**

http://www.diagnosticpathology.diagnomx.eu/vs/1194507146115753

## Background

Benign fibro-osseous lesions (BFOL) are a clinically diverse group of bone disorders that share similar histologic features and occur relatively commonly in the jaw. Common to all forms of BFOL is the replacement of normal bone with a tissue comprising collagen and fibroblasts containing varying amounts of mineralized substance, which may be bony or cementum-like in appearance. BFOL include developmental lesions, reactive or dysplastic processes, and neoplasms [1,2]. Ossifying fibroma (OF), fibrous dysplasia (FD), and osseous dysplasia (OD) are three forms of BFOL. The most common OF lesions including conventional and juvenile types that typically occur in the premolar-molar region of the mandible, with women more frequently affected [3]. OF is generally asymptomatic, but can cause serious cosmetic and functional problems [4]. Ossifying fibroma can present as a solitary lesion or rarely, as multiple lesions.

OF and FD show distinct patterns of disease progression, and it is important to distinguish between them. OF carries a risk of recurrence and must be completely enucleated from the surrounding bone. By contrast, FD growth usually stabilizes when skeletal maturity is reached; hence, surgical intervention is usually reserved for cosmetic or functional purposes [5-7]. However, these two lesions present diagnostic difficulties because of the uncertain significance of specific radiological and histological features, especially in biopsied specimens, and therefore, accurate diagnosis can be challenging. WHO has suggested three subtypes of fibrous dysplasia as follows: monostotic fibrous dysplasia (MFD), which involves only one bone; polyostotic fibrous dysplasia (PFD), which involves multiple bones; and McCune-Albright syndrome (MAS), which has at least two of the following triad: PFD, café-au-lait spots, and hyperfunctional endocrinopathy (such as precocious puberty, hyperthyroidism, growth hormone excess, and Cushing’s syndrome). Multiple bone involvement is more common in FD than in OF [2,8-10]. *GNAS* (guanine nucleotide-binding protein/α-subunit) mutations that induce the activation of G-protein α-subunit participate in the pathogenesis of fibrous dysplasia. There is a well-established association between fibrous dysplasia and post-zygotic activating mutations of the *GNAS* gene [11]. In a recent review by our group that updated the *GNAS* genetic mutation rate of in fibrous dysplasia was up to 86% (264/307), while no mutation was found in patients diagnosed with ossifying fibroma [9]. *GNAS* mutation detection may be helpful in differentiating fibrous dysplasia from other fibro-osseous lesions.

Hyperparathyroidism-jaw tumour syndrome (HPT-JT) is an autosomal dominant, multiple neoplastic syndrome primarily characterized by hyperparathyroidism caused by a parathyroid adenoma or adenocarcinoma [12,13]. Kidney lesions may also occur in HPT-JT, including bilateral cysts, renal hamartomas, or Wilms tumours. Benign and malignant uterine tumours are apparently common in women diagnosed with HPT-JT syndrome, including adenomyosis, adenofibroma, endometrial hyperplasia, leiomyoma, and adenosarcoma [14,15]. Recently, the candidate tumor suppressor gene *HRPT2* was identified in chromosome 1q24-q32, enconding a novel protein of 531 amionacids named parafibromin [16]. Some studies showed that alterations in *HRPT2* gene are related with HPT-JT and sporadic carcinoma and adenomas of parathyroid [9,17] Alterations in the tumor suppressor gene *HPRT2* in ossifying fibroma have recently been reported. Direct sequencing of the *HPRT2* revealed mutations in two out of the four cases of ossifying fibroma. However, one of the two mutational cases is HPT-JT syndrome because of the increased PTH level [18]. These findings indicate that the *HPRT2* mutation is not common in the development of sporadic ossifying fibroma, and therefore may not be used as a marker for diagnosis [5]. So when the *HRPT2* gene revealed mutations, the HPT-JT syndrome should be noticed. About 30–40% of individuals diagnosed with HPT-JT may also develop single or multiple OFs, which are distinct from the “brown” tumours associated with severe hyperparathyroidism [16,19,20]. Multiple OFs in patients diagnosed with HPT-JT have been reported, but the ratio remains unknown. Typically, OFs are encountered as solitary lesions in patients with no family history. Multiple ossifying fibromas are rare, and only a few cases have been reported. Here we report two cases of sporadic multiple OFs with an emphasis on differential diagnosis between FD and HPT-JT related OFs.

## Materials and methods

Cases diagnosed as OF during 1949–2013 were retrieved from the medical records of the Department of Oral Pathology, Peking University School of Stomatology. The study protocol was approved by the Ethical Committee for Human Experiments of Peking University School of Stomatology (IRB00001052-11041). Standard haematoxylin and eosin stained sections were reviewed, and the lesions were reclassified according to the WHO histological classification of odontogenic tumours [2]. Two cases of multiple OFs among 102 total confirmed OF cases were identified. Clinical data including age, sex, anatomic site, duration, radiographic features, clinical impression, treatment, and available follow-up data were reported. Mutational analysis of *GNAS* at the Arg201 and Gln227 codons and screening of all *HRPT2* exons were performed on the tissue samples of the 2 cases using direct sequencing. In addition, multiple OFs and HPT-JT related OF cases previously reported in the literatures were reviewed.

### Case presentation

#### Case 1

**Figure 1:**
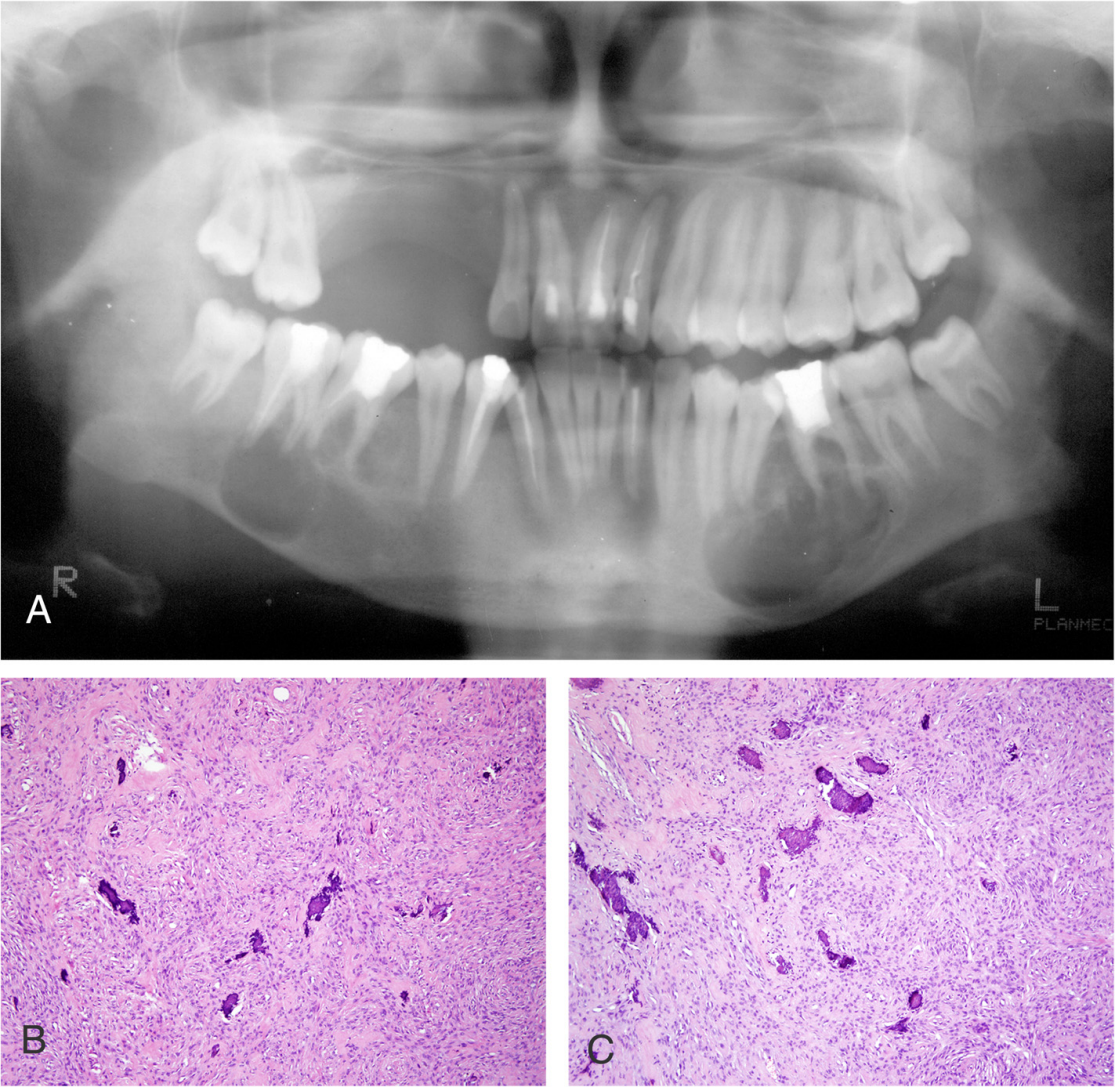
**Radiographic and histopathologic features of Case 1. (A)** Panoramic radiograph showing two well-demarcated radiolucencies bilaterally in the mandible. The right premolars were extruded and displaced, and the roots of two premolars were resorbed. **(B-C)** Haematoxylin and eosin stain (100×) showing interlacing bundles of collagen fibres and spindle cells associated with variably sized calcified tissue deposits.

The patient was a 19-year-old female, with a chief complaint of a slowly progressive, non-painful growth at the left mandible. Four years previously, maxillary surgery was performed at a local hospital on a lesion diagnosed as an ossifying fibroma. There was no specified anamnesis or family history of neoplasia. On present examination, clinicians observed a mild facial asymmetry with a hard swelling in the left mandible. The tumour was covered by normal intact alveolar mucosa. Panoramic radiography showed two well-demarcated bilateral radio lucencies in the mandible. The left premolars were displaced slightly due to extrusion, and the roots of two premolars were resorbed (Figure 1A). Biochemistry blood tests showed an elevated alkaline phosphatase of 278 IU/L (normal range 30–110 IU/L), but serum calcium and phosphorus were normal. PTH was not tested in this patient. The lesions were enucleated and easily from the surrounding bone. Histologically, the lesions demonstrated interlacing bundles of collagen fibres and cells associated with variable-sized calcified tissue deposits (Figure 1B-C). The combination of histopathologic, radiographic, and clinical features supported a diagnosis of sporadic multiple conventional ossifying fibroma. Mutational analysis of *GNAS* and *HRPT2* revealed no genetic abnormality in the lesion tissue samples. The lesions recurred one year after the first surgery, but the patient refused further treatment.

#### Case 2

**Figure 2:**
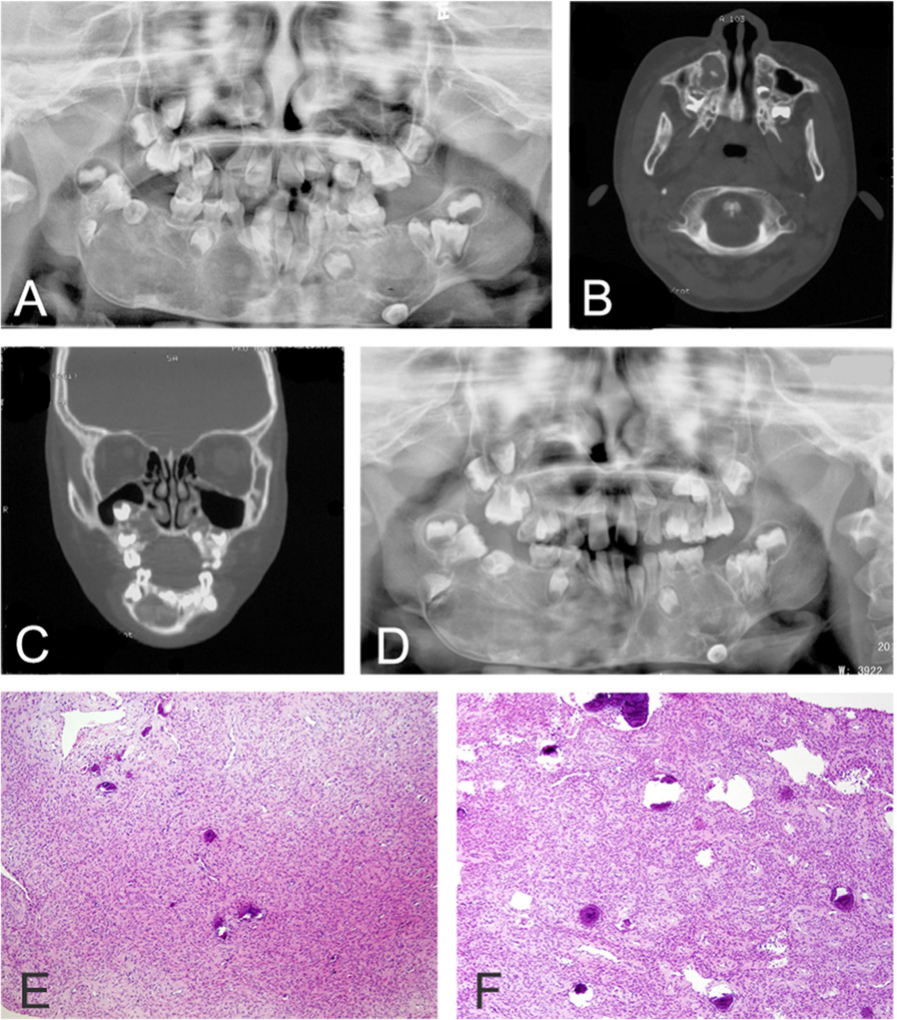
**Radiographic and histopathologic features of Case 2. (A)** Panoramic radiograph showing well-demarcated mixed-density lesions surrounded by sclerotic border involving four quarters of jaws. **(B-C)** CT scan demonstrating expansive lesions involving in the maxilla and mandible. **(D)** After 1 year, the lesions are visibly enlarged. **(E)** Haematoxylin and eosin stain (100×) of the mandibular lesion showing scarce areas of small spherical calcifications in dense fibroblasts. **(F)** Haematoxylin and eosin stain (100×) showing a similar maxillary lesion of densely packed fibroblasts and calcified structures.

A 6-year-old boy had synchronous lesions involving both the maxilla and mandible. His parents noticed the lesions 2 months prior to evaluation. The patient did not have any familial history of jaw disease. On examination, the lesions were firm and non-fluctuant, and bilateral mandibular buccal bone expansion was found extending from the deciduous canine to the deciduous molar. The overlying mucosa was intact. Panoramic radiography revealed a large lesion extending from the right ascending mandibular ramus to the left ascending mandibular ramus. The lesion consisted of a central heterogeneous mineralization and a thin marginal radiolucent area with a well-demarcated sclerotic border. Several tooth germs were displaced, and roots of multiple primary teeth were resorbed. The permanent molar teeth were significantly displaced, particularly the second molar, which was displaced into the ascending ramus (Figure 2A). A CT scan confirmed thinned bony cortex of the inferior mandibular border and alveolar bone expansion near the mixed radiopaque mass (Figure 2B). A displaced tooth germ grew into the maxillary sinus (Figure 2C). Blood tests (serum calcium, phosphorus, alkaline phosphatase, and PTH) were within normal limits. Incisional biopsies were performed in the mandibular and the maxillary lesions, and both specimens showed a similar histopathologic pattern. The lesions were mainly composed of fibrous tissue rich in fibroblasts with spherical calcifications. The mandibular lesion showed scarce areas of small spherical calcifications (Figure 2E); by contrast, the maxillary specimen exhibited a larger amount of these calcified structures (Figure 2F). The combination of histopathologic, radiographic, and clinical features supported a diagnosis of multiple conventional ossifying fibroma. Mutational analysis of *GNAS* and *HRPT2* revealed no genetic abnormality in the lesions. Because of the patient’s young age, the large size of the lesions, and the involvement of all four jaw quadrants, treatment was delayed, and the patient was closely followed up for 1 year (Figure 2D).

### Literature review of sporadic multiple ossifying fibroma

Based on a review of the literatures since 1968, together with the present two cases, 18 cases (16 previously reported cases [21-23]) of sporadic multiple ossifying fibroma were collected and summarized in Table 1. There were 13(72.2%) female and 5 (27.8%) male patients, and the mean age of the patients at initial diagnosis was 28.6 years old (range 6–55 years old). Bone swelling or expansion was the typical clinical sign and occurred in all patients. Two (11.1%) cases were found only in the mandible, and 4 (22.2%) cases were found only in the maxilla. Twelve (68.8%) cases were found in both the maxilla and mandible. Radiographic findings included unilocular and multilocular patterns demonstrating varying degrees of radiopacity in 5 cases (27.8%); radiolucencies with well-circumscribed borders were also found, and in 13 cases (72.2%), mixed density intralesional calcification was observed. Among the 14 cases providing therapeutic information, enucleation was the primary treatment in 11 cases, and recurrence was documented in 3 cases (3/14) after 6 months, 1 year, and 2 years, respectively. Extended resection with an interpositional bone graft/titanium was performed in 2 cases, and no recurrence was reported. Treatment in case 2 was delayed because of the patient’s young age, and the lesion was closely monitored during follow-up. In the previous 16 multiform OF cases, only 5 cases reported the serum calcium and phosphorus, and only 2 cases had special focus on the serum PTH.

**Table 1:** Summary of total 18 cases of sporadic multiple ossifying fibromas

**No.**	**Reference**	**Gender/on set age (year)**^**a**^	**Site**^**b**^	**Image features**^**c**^	**Displacement of the teeth/root Resorption**^**d**^	**Biochemistry test**^**e**^	**Treatment**	**Follow-up**	***GNAS *** **mutation analysis**	***HRPT2 *** **mutation analysis**
1	Bradley and Leake [38], 1968	F/6	Les. 1: rught maxilla Les. 2: right angle of the mandible	P.R.: multicystic Lesions	Yes/N.S.	N.S.	Right maxilla:enucleation and curettage right mandible: scheduled for removal	N.S.	N.S.	N.S.
2	Takeda and Fujioka [39], 1987	M/55	Les. 1: left maxilla Les. 2: right maxilla	P.R: well-circumscribed lesions showed radiolucent areas mixed with radiopaque areas	N.S.	N.S.	N.S.	Patient refused treatment	N.S.	N.S.
3	Hauser et al [40], 1989	M/35	Les. 1: right maxillary sinus Les. 2: left maxillary sinus	P.R.: well-circumscribed mixed radiolucent/radiopaque lesions CT: well-circumscribed lesion with calcified masses	No/N.S.	N.S.	Right maxillary sinus: enucleation left maxillary sinus: partial hemimaxillectomy	N.S.	N.S.	N.S.
4	Yih et al [41], 1989	F/31	Les. 1: left mandible Les. 2: right maxilla Les. 3: left mandible (2 years later)	P.R.: well circumscribed unilocular radiolucency	N.S.	ALP: 218 IU/L↑Ca : normal limits P: normal limits	Left mandibular body and right maxilla:enucleation	Recurrence of the left mandible after 2 y later	N.S.	N.S.
5	Khanna and Andrade [42], 1992	M/33	Les. 1: right maxilla Les. 2: left mandible	P.R.: large lesions contained diffused calcifications	N.S.	ALP: normal limits Ca: normal limits	Both of the two lesions: enucleation	Lost for follow-up	N.S.	N.S.
6	Hwang et al [43], 2001	F/25	Les. 1: right mandible Les. 2: left maxilla Les. 3: left mandibuar body Les. 4: left maxilla Les. 5: right maxilla	P.R.: large calcified mass surrounded by a radiolucent halo zone with corticated margin	Yes/yes	N.S.	Right mandible: partial hemimandibulectomy right maxilla:hemimaxillectomy	Initially refused treatment; 3 y later, surgical remission of the lesions was undertaken	N.S.	N.S.
7	Bertolini et al [44], 2002	F/37	Les. 1: left maxilla. and hard palate Les. 2: right mandible Les. 3: left mandible	P.R.: large radiolucency lesions with interspersed calcifications CT: revealed fibrous calcified masses that involved the left maxilla and the right and left mandibular body	No/no	N.S.	Right mandible: partial mandibulectomy Left mandible: curettage Left maxilla: intraoral surgical removal	Mandible: no recurrencey after 2 y Maxilla.: no recurrence after 1 y	N.S.	N.S.
8	Barberi et al [45], 2003	F/53	Les. 1: left infraorbital region Les. 2: right hard palate	P.R.: showed partial opacification of left maxillary sinus CT: two different multilocular, inhomogeneously hypodense entities walled in an irregularly thick sclerotic border	No /no	N.S.	N.S.	N.S.	N.S.	N.S.
9	Stergiou et al [46], 2007	F/36	Les. 1: left mandible Les. 2: right mandible Les. 3: left maxilla	P.R.: well circumscribed unilocular radiolucency containing diffuse calcifications CT: well demarcated lesions, low density and scattered calcifications	N.S.	N.S.	Enucleation and curettage	No recurrence after 6 months	N.S.	N.S.
10	Chindia et al [47], 2008	F/27	Les. 1: right angle and body of the mandible Les. 2: left maxilla	P.R.: mandibular lesion was corticated and maxillary lesion was less well defined with almost complete obliteration of the maxillary sinus	N.S.	N.S.	Both of the lesions: enucleation	Recurrence after 6 months (mandible)	N.S.	N.S.
11	Ribeiro et al [48], 2011	F/35	Les. 1: left mandible Les. 2: right mandible	P.R.: large radiolucency surrounded by a radiopaque halo in the left and right mandible CT: unilocular and hypodense image	Yes/yes	Ca: 9.73 mg/dl P: 4.2 mg/dL PTH: 56.34 pg/mL	Both lesions: enucleation	No recurrence after 3 y	N.S.	N.S.
12	Agarwal et al [50], 2012	F/20	Les. 1: left posterior maxilla Les. 2: right posterior mandible	P.R.: maxillary and mandibular lesion was well-defined with a radiolucent rim and sclerotic border CT: hyperattenuated masses in left maxillary and right mandibular alveolar ridges	Yes/no	N.S.	N.S.	Take an operation fifteen years ago of maxilla	N.S.	N.S.
13	Popli et al [21], 2012	F/19	Les. 1: left maxilla Les. 2: right mandible	P.R.: well-defined mixed radiolucent and radiopaque lesions CT: mixed-density, expansile lesions present at the alveolar process of both the maxilla walled by irregularly thick sclerotic border.	Yes/yes	No sign of hyperparathyroidism	Enucleation	No recurrence after 2 y	N.S.	N.S.
14	Akcam et al [49], 2012	M/20	Les. 1: left maxilla Les. 2: left mandible	P.R.: well defined,multilocular radiolucent lesion of the left mandible unilocular radiolucent lesion in the left maxilla CT: extensivehypodense lesions with cortical expansion	Yes/no	Ca, P, PTH within normal limits	Enucleation	No recurrence after 8 months	N.S.	N.S.
15	Kiran Desai et al [22], 2013	F/18	Les. 1: right maxilla Les. 2: right mandible	CT: large well-defined expansile lesion, heterogeneously hyper dense, multiple internal punctuate calcifications	Yes/N.S.	Blood calcium levels within normal limits, no sign of hyperparathyroidism	Enucleation	No recurrence after 2 years	N.S.	N.S.
16	Ponniah et al [23], 2013	F/45	mutiple lesions from the left mandible to the right mandible	P.R.: multilocular radiolucent lesion CT: osteolytic, soft-tissue density lesion with thinning and erosion of the buccal cortex in the anterior region of the mandible	Yes/yes	N.S.	Enucleation	No recurrence after 5 months	N.S.	N.S.
17	Present case 1	F/15	Les.1: right maxilla Les. 2, 3: bilateral mandible	P.R.: well demarcated radiolucency in the bilateral mandible	Yes/yes	Ca: 2.7 mmol/L P: 0.77 mmol/L↓ ALP: 278 IU/L↑, PTH not find to text	Enucleation	Recurrence after 1 year	No	No
18	Present case 2	M/6	Les. 1: right maxilla Les. 2: left maxilla Les. 3: right mandible Les. 4: left mandible	P.R.:central inhomogeneous mineralizationin thin marginal radiolucent area with a well demarcated sclerotic border CT: mixed radiopaque image	Yes/yes	Ca: 2.63 mmol/L P: 1.58 mmol/L ALP: 62 IU/L PTH 9.12 pg/mL	Incisional biopsies the treatment delayed because of the young age	After 1 year, the lesions enlarged obviously	No	No

^a^F = female; M = Male.

^b^Les. = lesion.

^c^P.R. = panoramic radiograph; CT = Computerized Tomography.

^d^N.S. = not stated.

^e^ALP = Alkaline phosphatase; Ca = Serum calcium; P = Phosphate.

### Literature review of HPT-JT related OF

In total, 24 cases of HPT-JT related ossifying fibroma were reviewed and are summarized in Table 2[12,15,24-37]. Of the 20 cases providing patient age and gender information, 6 patients were female, and 14 were male. The mean age at initial diagnosis was 23.8 years old (range 13 years to 54 years). Sixteen (66.7%) patients were diagnosed with a single lesion, and 8 patients (33.3%) were diagnosed with multiple jaw lesions. Among the 16 cases of unilocular lesions, 12 cases were located in the mandible, and 4 were in the maxilla; of the 8 multilocular cases, 2 were found only in the mandible, 1 case was only in the maxilla, and 5 cases were found in both the maxilla and the mandible.

**Table 2:** Cases of ossifying fiboma affected with HPT-JT in the literature

**Number**	**Author**	**Age/gender**^**a**^	**Single/multiple**	**Location**
1	Kutcher [24]	22/M	Single	The right posterior mandible
2	Iacobone [25]	26/F	Single	Left mandible ramus
3	Yamashita [15]	18/M	Single	Right maxilla
4	Raue [26]	29/M	Single	Mandible
5	Moon [27]	18/M	Single	Left mandible
6		17/F	Single	The right mandible
7	Teh [28]	26/M	Single	Left maxilla
8	Mallette [29]	36/N.S.*	Single	Maxilla
9	Rekik [30]	23/F	Single	The right body of the mandible
10	Dinnen [31]	18/M	Single	The right molar region of the mandible
11	Cavaco [32]	18/M	Single	Maxilla
12		31/M	Single	Mandible
13		23/M	Single	Mandible
14		21/F	Single	Mandible
15	Wamakulasuriya [36]	37/M	Single	The left mandible
16		43/M	Single	The left mandible
17	Teh [28]	54/M	Multiple	Lesion 1: mandible
				Lesion 2: maxilla
18	Mallette [29]	17/N.S.*	multiple	Lesion 1: mandible
				Lesion 2: hard palate
19	Schmidt [33]	37 M	Multiple	Lesion 1: right maxillary canine and premolar areas
				Lesion 2: left maxillary canine and premolar areas
				Lesion 3: left mandible
				Lesion 4: right mandible
20	Szabo [34]	22/M	Multiple	Lesion 1: right maxilla
				Lesion 2: in the right mandible
21	Howell [35]	16/M	Multiple	Lesion 1: right mandible
				Lesion 2: right mandible
22	Aldred [12]	22/M	Multiple	Lesion 1 the right mandible
				Lesion 2: the left mandible
23	Fujikawa [37]	22/F	Multiple	Lesion 1: left maxilla
				Lesion 2:left mandible
				Lesion 3: right mandible
24	Cavaco [32]	13/F	Multiple	Lesion1: maxilla
				Lesion 2: mandible

^a^Age at first presentation, F = female, M = male, *N.S. = not stated.

## Discussion

The ratio of multiple lesions OF cases to all OF cases is unclear, but we found only 2 cases in 102 case reviews (2.0%). Only 16 known cases of multiple ossifying fibroma have been reported (Table 1) [21-23,38-50]. Although the patients were young in our two reported cases, the cases are not considered juvenile ossifying fibromas (not psammomatoid or trabecular type) because of the histologic features. The etiology and pathogenesis for both solitary and multiple OF remain unknown. However, both forms of OF present very similar clinical, radiologic, and histopathologic features, suggesting that they are different clinical presentations of the same disease [48].

According to the latest WHO classification, ossifying fibroma and fibrous dysplasia can be distinguished in the following manner. Radiologically, ossifying fibroma is a well-demarcated lesion and does not merge with the surrounding bone. Histopathologically, normal bone is replaced by fibroblastic stoma with calcifications and osteoblastic rimming observed [2,51]. However, the histopathologic characteristics of the two diseases may overlap, making diagnosis difficult. Detection of *GNAS* mutations is one valuable diagnostic adjunct [9]. Somatic mutations at the Arg^201^ and Gln^227^ codon of Gsα have been identified in many fibrous dysplastic lesions, but are absent in ossifying fibromas, which points to a possible role for mutational analysis in differentiating these two conditions [5,9,52,53]. In the present two cases, the lesions had a demarcated border radiographically, as well as tooth displacement and root resorption. Genetic screening of the *GNAS* gene at Arg201 and Gln227 codon did not show any anomalies, which confirmed the diagnosis of OF.

HPT-JT, considered a rare variant of familial HPT, was first described in 1990 [14]. Hypercalcemia and high PTH levels are associated with HPT-JT, andossifying fibromas reportedly occur in 25–50% of HPT-JT cases [16,54]. We reviewed the literature reporting cases of HPT-JT with jaw ossifying fibromas (Table 2). In the 24 identified cases, 16 (66.7%) cases were solitary lesions, and 8 (33.3%) cases were multiple lesions. Thus, sporadic multiple ossifying fibroma must be distinguished from cases of HPT-JT. HPT-JT has been classically described as a more aggressive disease characterized by multiple organ involvement (45%–75%), increased risk of persistence and recurrences (20%–50%), and parathyroid carcinoma (10%–40%) [25]. The suggested therapeutic approach is parathyroid gland resection, which prevents parathyroid carcinoma recurrence [55,56]. Differential diagnosis of ossifying fibroma associated with HPT-JT from sporadic ossifying fibroma is important for treatment and prognosis. In the cases reviewed, the serum calcium and PTH levels were normal in case 2, allowing us to exclude the association with HPT-JT. PTH was not measured in case 1, but the patient had a normal calcium level and no family history of HPT-JT. Therefore, both cases were excluded from an association with HPT-JT. In the previously reported 16 multiform OF cases, only 5 cases had reported serum levels of calcium and phosphorus, and only 2 cases reported the serum PTH level; therefore, an association with HPT-JT cannot be ruled out. *HRPT2* genetic mutations are associated with the hereditary pathogenesis of HPT-JT syndrome [16]. Thus, *HRPT2* genetic evaluation was conducted in the present two cases, and showed no mutational alternation. Based on the clinical and molecular findings, these two cases should be considered sporadic, non HPT-JT cases.

There is another heterogeneous group of reactive BFOL lesions, known as osseous dysplasia (OD); the lesions are associated with the tooth apex and may be unifocal or florid, involving most of the mandible [57]. The 2005 WHO classification divides ODs into focal, periapical, and florid OD, and familial gigantiform cementoma [2]. Differential diagnosis between ossifying fibroma and osseous dysplasia is important. Radiographically, OD is diffuse and amorphous, with mixed radiopaque to radiolucent lesions. Histologic features include a cellular connective tissue stroma punctuated by irregular osseous and/or cementum-like calcifications [58]. According to the authors [58], the criteria distinguishing OD from other fibro-osseous lesions are: (1) a histologic pattern consisting of cellular mesenchymal tissue with intermixed calcifications; (2) radiolucent and/or radiopaque lesions in the jaws; (3) surgically, an easily fragmented, haemorrhagic, gritty mass difficult to remove from the bone; and (4) gross observations of multiple haemorrhagic fragments of variable consistency. Osseous dysplasia is thought to be non-neoplastic and originating from the periodontal ligament [3]. Further surgical intervention is not necessary for small lesions, but periodic follow-up is recommended. Most OFs were not associated with the tooth apex, but often caused divergence or displacement of involved teeth [59]. Another rare hereditary condition with radiographic and histologic features similar to florid OD is familial gigantiform cementoma, which tends to occur in early childhood or teenhood [10]. Exuberant fibro-osseous lesions occurring in multiple jaw quadrants were designated as gigantiform cementomas or familial multiple cementomas in the first edition of the WHO’s Histological Typing of Odontogenic Tumours, Jaw cysts, and Allied Lesions [60]. These lesions have also been reported as familial florid cemento-osseous dysplasia [61] and familial florid osseous dysplasia [62]. Although cases with a familial pattern are noted in a few publications, sporadic cases without a family history have also been reported [63]. Some authors suggested that these cases were classified primarily as “ossifying fibroma” rather than “gigantiform cementoma” [64]. Compared to the multiple ossifying fibroma, the clinicoradiologic features are similar to those of florid osseous dysplasia: lesions surrounding the root; an outer layer of dense opacities, and multiquadrant, expansive, mixed radiolucent to opaque lesions crossing the jaw midlines. Microscopically dispersed throughout the lesions are ovoid, often laminated, variably sized psammomatoid calcifications. Many of these spheroidal calcifications are large, much larger than those observed in the psammomatoid variant of ossifying fibroma [1]. Under polarized light, Sharpy’s fibres are seen projecting radially from these larger spheroidal deposits and resemble cementicles normally encountered in the periodontal ligament [1].

Fibro-osseous lesions of the jaw and face must be differentiated from other bone lesions that may mimic them histologically and radiographically. The most important lesions in differential diagnosis are osteoblastoma, adamantinoma and giant cell granuloma. Osteoblastoma is a benign radiolytic bone-forming neoplasm that is most common in the postcranial skeleton, particular the posterior elements of the spine, and it also may occur in the maxillofacial region [65]. Histologically, the central differentiating characteristic is the lack of cellular spindle cells in the stroma, which instead comprises loose vasculature with numerous prominent epithelioid-type osteoblasts [57]. Nawal Hammas’ recent study shows that P63 may serve as a biomarker for the differential diagnosis between giant cell tumor of bone and other morphologically similar lesions, especially central giant cell granuloma since the latter does not express P63 [66]. Adamantinoma is a primary low-grade, malignant bone tumor that is predominantly located in the mid-portion of the tibia. Histologically, classic adamantinoma is a biphasic tumor characterized by epithelial and osteofibrous components that may be intermingled with each other in various proportions and differentiating patterns [67]. Chondromyxoid fibromas are rare benign chondroid/myxoid matrix-producing tumors that occur in metaphyses of long tubular bones. Prior cytogenetic analyses have identified complex abnormalities involving chromosome 6 in the majority of cases and the cells can be positive for actin [68].

## Conclusion

In conclusion, the suggested therapeutic approach for HPT-JT is parathyroid gland resection to prevent occurrence of the parathyroid carcinoma. Differential diagnosis of ossifying fibroma associated with HPT-JT and sporadic ossifying fibroma is important for treatment and prognosis. We presented 2 cases of multiple ossifying fibroma to illustrate to clinicians and radiologists that ossifying fibroma may have multiple forms and can be linked to hereditary syndromes. Genetic screening of *GNAS* could facilitate differential diagnosis of fibrous dysplasia at the molecular level. Furthermore, we strongly recommend that patients diagnosed with multiple or familial ossifying fibromas receive serum tests for PTH and mutation screening of *HRPT2*, which could exclude possible association with the HPT-JT syndrome.

## Consent

Written informed consent was obtained from the patient (patient 1) and the patient's parent (patient 2) for publication of this Case Report and any accompanying images. A copy of the written consent is available for review by the Editor-in-Chief of this journal.

## Abbreviations

BFOL: Benign fibro-osseous lesions; OF: Ossifying fibroma; FD: Fibrous dysplasia; OD: Osseous dysplasia; MFD: Monostotic fibrous dysplasia; PFD: Polyostotic fibrous dysplasia; HPT-JT: Hyperparathyroidism-jaw tumour syndrome; PTH: Parathyroid hormone.

## Competing interests

The authors declare that they have no competing interests.

## Authors’ contributions

TTW and RZ participated in the histopathological evaluation, performed the literature review, acquired photomicrographs and drafted the manuscript. YC and QD established the diagnosis of the case described in figures and performed the radiological examination. LW and TJL conceived and designed the study, and revised the manuscript for important intellectual content. All authors read and approved the final manuscript.

## Authors’ information

Ting-ting Wang and Ran Zhang: co-first author.
